# Photon skyshine from medical linear accelerators

**DOI:** 10.1002/acm2.12833

**Published:** 2020-03-01

**Authors:** Patrick N. McDermott

**Affiliations:** ^1^ Beaumont Health System Royal Oak MI USA

**Keywords:** accelerator shielding, linac, medical accelerator, photon skyshine

## Abstract

A widely used formula for the prediction of photon skyshine has been shown to be very inaccurate by comparison with numerous measurements. Discrepancies of up to an order of magnitude have been observed. In addition to this, the formula does not predict the observed dependence on field size, nor the fact that skyshine dose rates exhibit a local maximum. A scaling formula is derived here, with a single fitting parameter, which properly accounts for these properties, provides physical insight into the skyshine phenomenon, and is more accurate. The location of the maximum dose rate depends on the ratio of the roof height above isocenter to the distance from the isocenter to the outer surface of the sidewall. For nominal linac room dimensions, the maximum dose occurs at a distance from the outer wall of approximately two times the height of the roof above the isocenter. The skyshine dose rate is proportional to the field area and not Ω^1.3^, as predicted by the standard formula, where Ω is the solid angle subtended by the beam. For lightly shielded roofs (concrete thickness less than about 0.5 m), the photon skyshine for 6 MV exceeds that for 18 MV. Evidence is presented that at intermediate distances the skyshine declines as one over the distance and not one over the distance squared. Predictions of skyshine dose rates depend critically on accurate knowledge of the roof transmission factor. If a roof is shielded so as to avoid designation as a “high radiation area,” photon skyshine will be negligible.

## 1 INTRODUCTION

Skyshine is radiation scattered to outdoor ground level by air above the roof of a radiation facility. This will be most important when the gantry is pointed upward. If the roof has little or no added radiation shielding “a problem may then arise as a result of the radiation scattered by the atmosphere to points at ground level outside the treatment room.”^1^ A widely quoted formula is given in National Council on Radiation Protection (NCRP) Report No. 151, “structural shielding design and evaluation for megavoltage x‐ and gamma‐ray radiotherapy facilities,” for the evaluation of the photon dose equivalent rate of skyshine.^1^ This formula has been acknowledged to be very inaccurate in a number of publications.^1^, ^2^, ^3^, ^4^, ^5^ In this paper, measurements that have been made of photon skyshine at various medical linac facilities will be analyzed to extract some of the properties of skyshine. A scaling relation will be derived that fits the measured data far better than the equation quoted in NCRP 151 and, in addition, provides physical insight into the skyshine phenomenon. It will be shown that, even with moderate roof shielding, photon skyshine is negligible.

## 2 MATERIALS AND METHODS

**Figure 1:**
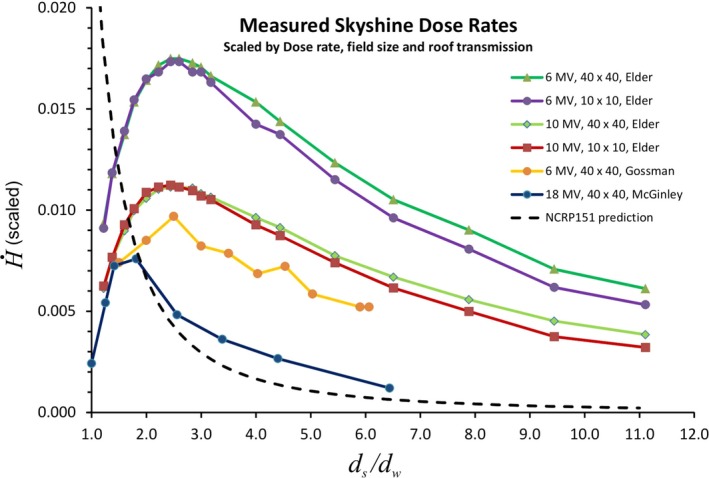
Scaled equivalent skyshine dose rates for 6, 10, and 18 MV and for various field sizes as a function of the scaled distance from the isocenter. The dose rates have been scaled by dividing the dose rate in nSv/s by the product of the dose rate at isocenter, the roof transmission factor and the field area at isocenter. The distance has been scaled by dividing by the distance to the outside surface of the side wall of the linac vault. Scaling allows easier comparison of this disparate data. The NCRP151 prediction is based on Eq. (1). See text for more detail.

**Figure 2:**
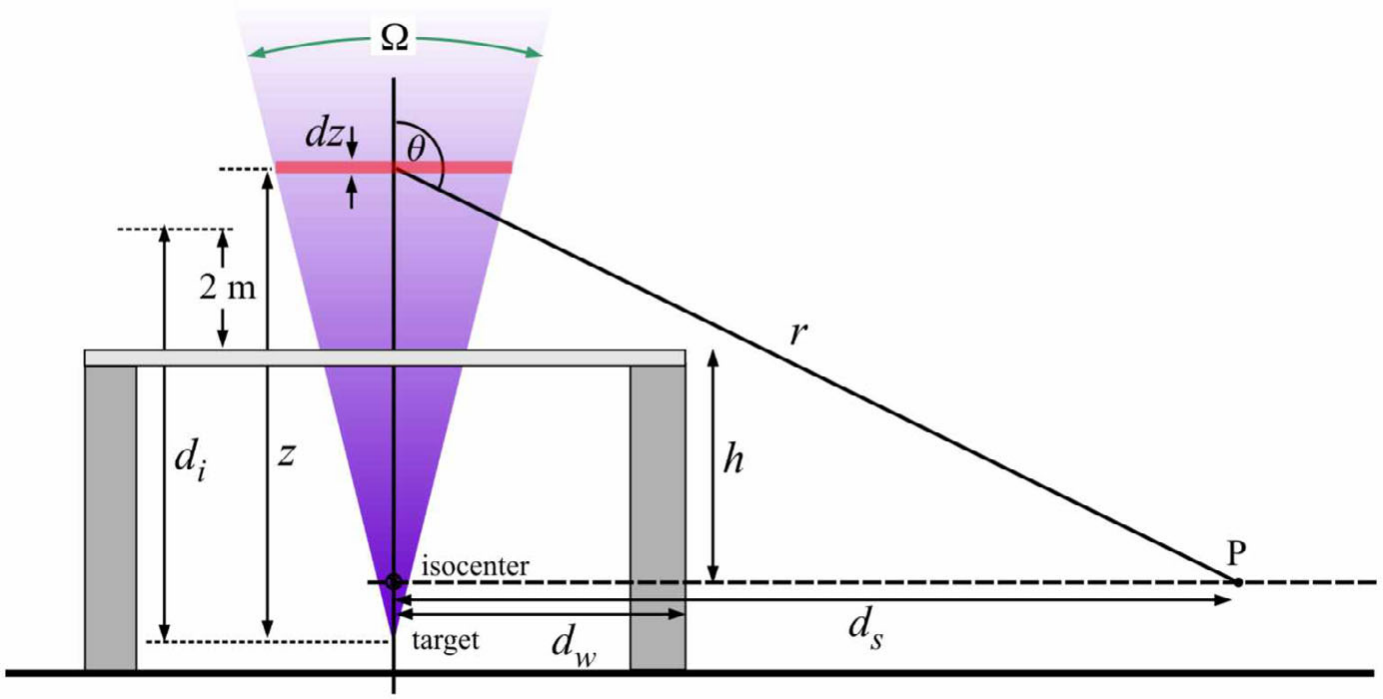
The geometry of skyshine from a medical linac vault. The scattering angle is *θ* (at height *z*) and Ω is the solid angle subtended by the beam. The observation point at distance *d_s_* is labeled P.

Measurements of medical linac photon skyshine radiation levels have been made by McGinley, Gossman, et al, de Paiva and da Rosa, Elder, et al and Rostampour, et al.^2^, ^3^, ^4^, ^5^, ^6^ Figure 1 shows a graph of some of these data. For purposes of comparison, the measured skyshine equivalent dose rates, in nSv/s, have been scaled by dividing them by the product ${\dot{D}}_{0}\;B_{\mathrm{xs}}\left(F_{0}\;/100{\;\text{cm}}^{2}\right)$, where ${\dot{D}}_{0}$ is the dose rate (in cGy/min) at the isocenter, *F*_0_ is the field area (in cm^2^) at the isocenter and *B_xs_* is the reported roof transmission for the facility and beam energy. The equivalent dose rates, $\dot{H}$(hereafter dose rate), are plotted as a function of *d_s_*/*d_w_*, where *d_s_* is the distance from the isocenter to the point of measurement and *d_w_* is the distance from the isocenter to the outer surface of the linac vault side wall (see Fig. 2).

Skyshine parameters from some of the references cited above can be found in Table 1. The quantity *d_i_* is the distance from the target to a point 2 m above the top surface of the roof (see Fig. 2), *h* is the distance from the isocenter to the top surface of the roof and *d_max_* is the distance from the isocenter at which the skyshine dose rate has its maximum value.

**Table 1:** Skyshine parameters.

Authors	Beam energy (MV)	Field size *F*_0_ (cm^2^)	${\dot{D}}_{0}$(cGy/min)	*d_i_* (m)	*d_w_* (m)	*h* (m)	*B_xs_*	(*d_ma_*_x_ − *d_w_*)/*h*
Elder, et al.	6	10 × 10	600	6.6	4.5	3.6	0.027	1.9
	6	40 × 40						1.9
	10	10 × 10					0.052	1.8
	10	40 × 40						1.8
Gossman, et al.	6	40 × 40	400	5.3	3.0	2.3	0.038	2.0
McGinley (NCRP 151)	18	40 × 40	400	6.0	7.5	3.0	0.9 (1.0)^a^	2.0

a Conflicting statements regarding this value are found in NCRP151 and McGinley.

Figure 1 shows that $\dot{H}$ rises rapidly with distance *d_s_* just beyond the outside wall, reaches a peak, and then declines with increasing distance. This implies that survey measurements should not just be made at a distance of 30 cm beyond the outer barrier, as for radiation transmitted through the side wall, but at distances of up to 15 m.^1^ It has been stated in the literature that the maximum dose rate occurs at a distance from the outer surface of the side barrier about equal to the height of the barrier.^1^, ^5^ The data in the last column of Table 1 show that, to a good approximation, the maximum actually occurs at a distance from the outer wall surface of approximately *twice* the distance from the isocenter to the roof surface. Gossman, et al state that the value of *d_max_* depends on field size but this is not apparent in the Elder et al data.^2^, ^4^ It will be shown below that the location of the maximum dose is expected to depend on the ratio *h*/*d_w_*.

With the exception of the Gossman et al. data, the scaled 6 MV $\dot{H}$ is largest followed by 10 MV and then 18 MV. The physical interpretation of this will be discussed later in this paper. For the Gossman data, the *calculated* value of *B_xs_* has been used for scaling, whereas for the Elder data the value of *B_xs_* was actually measured.^2^, ^4^ The Gossman value of *B_xs_* is based on the calculated transmission for 0.51 m of concrete. This ignores any other material that may be in the ceiling and roof structure. The presence of as little as 3 cm of steel (to support the 0.51 m of concrete) at 6 MV, would reduce *B_xs_* by a factor of 2 and then the scaled measurements would fall right on top of the 6 MV Elder data. In addition to this, there is a statement in NCRP 151 regarding tenth value layer (TVL) values that “Concrete values are based on a conservatively safe adaptation from Nelson and LaRiviere …^1^” This implies that calculations of barrier transmission *B_xs_* may be conservatively high. Accurate predictions of skyshine intensity depend critically on the accuracy of the roof transmission factor.

Linac photon skyshine predictions have been based on a widely quoted empirical equation that is reproduced here for reference.^1^, ^2^, ^3^, ^4^, ^5^, ^6^ The geometry is shown in Fig. 2. The dose equivalent rate in nSv/h at a distance *d_s_* (in meters) from the isocenter is:

(1) $$\dot{H}=2.5\times 10^{7}\frac{B_{\mathrm{xs}}\;{\dot{D}}_{0}\;\Omega^{1.3}}{{\left(d_{i}\;d_{s}\right)}^{2}},$$

where, *B_xs_* = roof shielding transmission factor for photons; Ω = the solid angle subtended by the beam (in steradians); ${\dot{D}}_{0}$ = x‐ray absorbed dose rate at a distance of 1.0 m from the target (Gy/h); *d_i_* = the vertical distance from the x‐ray target to a point 2 m above the top of the roof (in meters); *d_s_* = distance from the isocenter (in meters).

The solid angle Ω subtended by a square field of side length *a* (at a distance of *h_i_* from the source) is:^7^

(2) $$\Omega =4\sin^{-1}\left(\frac{a^{2}}{a^{2}+4h_{i}^{2}}\right).$$

This reduces to the expected value when *h_i_ *>> *a*, namely Ω ≈ (*a*/*h_i_*).^2^ Much has been made in the literature about this, but as the solid angle is small, the approximation stated in the previous sentence is accurate to within about 4% even for a 40 × 40 cm^2^ field.^2^, ^3^, ^6^, ^7^ According to McGinley, Eq. (1) is based on measurements made near Cs‐137 and Co‐60 sources placed in a hole in the ground.^5^

Equation (1) predicts that $\dot{H}$ is proportional to Ω^1.3^. The data in Fig. 1 have been scaled by dividing by the field area *F*_0_ (in cm^2^). It can be seen that for the Elder data, the scaled graphs for a 10 × 10 cm^2^ field size lie nearly on top of the data for 40 × 40 cm^2^. This is evidence that $\dot{H}$ is directly proportional to field area. This is consistent with Fig. 2 of Gossman, et al in which they plot the dose rate as a function of the side length of the field for various distances *d_s_*. These authors state that “the atmospheric scattering relationship is a function of field size to some second order magnitude polynomial.^2^” For the Elder data, the ratio of the exposure rates at *d_max_* for a 40 × 40 cm^2^ field to that for a 10 × 10 cm^2^ field is 16.3 for 6 MV and 16.1 for 10 MV.^4^ This is strong evidence that $\dot{H}$ is proportional to the field area. It will be shown below that this is expected based on physical arguments. Equation (1) predicts that the ratio should be (Ω_40_/Ω_10_)^1.3^ ≈ (4)^2.6^ = 37, this is clearly not correct.

Equation (1) predicts that $\dot{H}\propto 1/d_{s}^{2}$. Fits to a power law ($1/d_{s}^{n}$) of the Elder et al data in Fig. 1 for *d_s_ *> 19 m, show that for 40 × 40 cm^2^, *n* = 0.95 ± 0.03 for 10 MV and *n* = 0.95 ± 0.05 for 6 MV. The quoted uncertainties are based on 1 standard deviation. For 18 MV (40 × 40 cm^2^, *d_s_ *> 19 m), fits to the McGinley data show that *n* = 1.5 ± 0.2. For the 6 MV, 40 × 40 cm^2^ Gossman et al data with *d_s_ *> 10 m, *n* = 0.77 ± 0.12. These data show that $\dot{H}$ does not obey an inverse square law within a distance of 50 m. The formula derived below predicts that *n* = 1.0.

It has been established in numerous references that the photon skyshine predicted values based on Eq. (1) are in serious error.^1^, ^2^, ^3^, ^4^, ^5^, ^6^ This is acknowledged within NCRP 151 in Table 5.1 of that document, which lists predicted values and values measured by McGinley for an 18 MV, 40 × 40 cm^2^ field (440 cGy/min) as a function of distance from the isocenter: “there is very poor agreement between the calculated and measured values.^1^” The calculated values are too high by a factor of 4 for small *d_s_* and too low by a factor of about 5 for distances of about 50 m. Gossman et al state that the discrepancies are as large as an order of magnitude for a 6 MV, 10 × 10 cm^2^ field.^2^ Scaled predicted values based on Eq. (1) are plotted in Fig. 1 for a 40 × 40 cm^2^ field. Nominal values have been chosen for *d_w_* (=5 m) and *d_i_* (=6 m) (see Table 1). It can be seen that Eq. (1) does not even reproduce the qualitative features of the measurements and is grossly in error at *d_max_* except for 18 MV.

In view of the poor predictive value of Eq. (1), it is desirable to find a simple, approximate scaling law for the dose rate for skyshine photons as measured at a distance *d_s_* (shown in Fig. 2) from the isocenter.^2^ The number of photons scattered per unit time toward a detector subtending solid angle ΔΩ is given by:

(3) $$\Delta {\dot{N}}_{s}=n\;\dot{\Phi }\frac{d\sigma }{d\Omega }\Delta \Omega ,$$

where *n* is the number of scattering centers, $\dot{\Phi }$ is the fluence rate (number of incident photons per unit area per unit time) and *d*σ/*d*Ω is the differential cross section for Compton scattering.^8^ Let us compute the contribution from a scattering volume element that is a cross section of the beam with thickness *dz* as shown in Fig. 2. In this case, *n* = *ρ_e_ F*(*z*) *dz*, where *ρ_e_* is the electron density (per unit volume) of the air, *F* is the beam cross sectional area at distance *z* from the target. *F*(*z*) = *F*_0_ *z* ^2^, where *F*_0_ is the field size (area) at the isocenter. $\dot{\Phi }\left(z\right)=B_{\mathrm{xs}}\left({\dot{\Phi }}_{0}/z^{2}\right)$, where ${\dot{\Phi }}_{0}$ is the fluence rate at the isocenter (1.0 m from the source), *z* is measured in meters from the target and *B_xs_* is the transmission through the roof. The dose rate at the isocenter ${\dot{D}}_{0}$ can be expressed in terms of the fluence rate as ${\dot{D}}_{0}=E_{\gamma }{\dot{\Phi }}_{0}\left(\mu_{\mathrm{en}}/\rho \right)$ where we assume monoenergetic photons of energy *E_γ_* and mass‐energy attenuation coefficient $\left(\mu_{\mathrm{en}}/\rho \right)$ for water. We assume that the transmission factor *B_xs_* is relatively high so that most of the photons passing through the roof are unscattered. It is also assumed that the air does not significantly contribute to attenuation of the primary beam. For a 6 MV primary beam, the average photon energy is approximately 2 MeV. The linear attenuation coefficient for 2 MeV photons in dry air at sea level is about 5.3 × 10^−5^ cm^−1^ and therefore the mean free path (1/*μ*) is on the order of 200 m. It is assumed that all scattering is from the beam central axis in order to simplify the geometry. This is reasonable to the extent that the radius of the disk in Fig. 2 is small compared to the distance from it to the point of observation. In addition, we ignore multiple scattering.

The photon fluence rate reaching the detector at point P in Fig. 2 due to scattering from the volume element of thickness *dz* is ${\dot{\Phi }}_{P}=\left(\Delta {\dot{N}}_{P}/\Delta A\right)=n\left(z\right)\;\dot{\Phi }\left(z\right)\left(d\sigma /d\Omega \right)\left(e^{-\mu^{\prime }r}/r^{2}\right)$, where *r* is the distance from the scattering element to the point of observation and *μ*′ is the linear attenuation coefficient for the scattered photons (assumed monoenergetic). The photons are scattered at angles between 90° and 180° (see *θ* in Fig. 2). For primary photons of energy *E*_γ_ >> *m*_0_ *c* ^2^ = 0.51 MeV, the scattered photons will have energy 0.51 MeV for scattering at 90° and 0.25 MeV for scattering at 180°. Photons of energy 0.25 MeV have a mean free path of about 72 m in air. We therefore neglect absorption or scattering of the *scattered* photons as we are interested in distances *d_s_* of about 20 to 50 m or less.

The dose rate at point P due to the fluence rate reaching point P is $\dot{H}=E_{\gamma^{\prime }}{\dot{\Phi }}_{P}\left(\mu_{en-s}/\rho \right)$ where $E_{\gamma^{\prime }}$ is the energy of the scattered photons and $\left(\mu_{en-s}/\rho \right)$ is the mass‐energy absorption coefficient of the scattered photons.

We assume that the side walls of the structure are completely opaque to skyshine radiation. This seems valid given the low energy of these photons. With this assumption, the only scattered photons that can reach point P must originate at some minimum distance above the roof. The minimum scattering angle at a distance *d_s_* from isocenter is:

(4) $$\theta_{m}=\frac{\pi }{2}+\tan^{-1}\left(\frac{h}{d_{s}-d_{w}}\right).$$

The skyshine is calculated at the vertical height of the isocenter, which is usually 1.3 m. For the differential cross section *dσ/d*Ω, the scattering angle is between 90° and 180°, we assume that *E*_γ_ >> *m*_0_ *c* ^2^ even though this assumption is marginal at low energies. Under these circumstances *dσ/d*Ω ≈ [*α* (1 − cos *θ*)]^−1^where *α = E*_γ_/*m*_0_ *c* ^2^. The average photon energy from linac bremsstrahlung is the nominal accelerating potential in MV divided by 3. For a 6 MV beam we thus have *α* ≈ 4.^3^

Putting all of the pieces together, the contribution to the dose equivalent rate $d\dot{H}$ from a scattering element of thickness *dz* is:

(5) $$d\dot{H}=\left(\frac{\mu_{en-s}/\rho }{\mu_{\mathrm{en}}/\rho }\right)\rho_{e}F_{0}\;B_{\mathrm{xs}}\;{\dot{D}}_{0}\frac{E_{\gamma^{\prime }}}{E_{\gamma }}\frac{d\sigma }{d\Omega }\frac{\mathrm{dz}}{r^{2}}.$$

The total instantaneous dose equivalent rate can be written in terms of the scattering angle *θ*:

(6) $$\dot{H}=\frac{1}{2}r_{e}^{2}\left(\frac{\mu_{en-s}/\rho }{\mu_{\mathrm{en}}/\rho }\right)\rho_{e}F_{0}\;\;B_{\mathrm{xs}}\;\;{\dot{D}}_{0}\frac{1}{\alpha^{2}d_{s}}\int_{\theta_{m}}^{\pi }\frac{d\theta }{{\left(1-\cos\theta \right)}^{2}}.$$

where *r_e_* is the classical electron radius. In principle, the mass attenuation coefficient for scatter, should remain inside the integral as it depends on the angle of scattering.

## 3 RESULTS AND DISCUSSION

Carrying out the integration in Eq. (6), and scaling the field size to 20 × 20 = 400 cm^2^ and the dose rate to 400 cGy/min at isocenter results in:

(7) $$\dot{H}=k\left(\frac{F_{0}}{400}\right)B_{\mathrm{xs}}\left(\frac{{\dot{D}}_{0}}{400}\right)\frac{1}{d_{s}}\left[2{\left(1+x^{2}\right)}^{3/2}-x\left(2x^{2}+3\right)\right],$$

where $\dot{H}$ is the instantaneous dose rate in units of nSv/s, *k* is an energy dependent proportionality constant, *F*_0_ is the field area at isocenter expressed in cm^2^, ${\dot{D}}_{0}$ is the dose rate at isocenter expressed in cGy/min, *d_s_* is the distance from the isocenter to the point of observation in meters and *x* = *h/*(*d_s_* – *d_w_*).

The rise in dose rate for points just beyond the side wall is primarily due to the fact that the distance *r* from the minimum observable altitude (of the point of scatter) to the observation point (see Fig. 2) is large at first and then drops rapidly with increasing *d_s_* (as *d_s_* → *d_w_*, *r* → ∞). As *d_s_* increases further, *r* begins to increase. This local maximum is not due to partial transmission through the roof or side wall or primarily to a higher probability of scatter at smaller angles. We have assumed that the side walls are opaque to photons scattered by air. In addition, the probability of scatter only rises very slowly with decreasing scatter angle for large angles.

Let us contrast Eq. (7) with Eq. (1). Equation (1) has a 1/$d_{s}^{2}$ dependence. The dependence of Eq. (7) on *d_s_* is somewhat complex but in the limit that *d_s_ *>> *d_w_* and *h*, the dependence is 1/*d_s_* and not 1/$d_{s}^{2}$. This is loosely analogous to the electric field around an infinite line charge in electrostatics, which is inversely proportional to the distance from the line. If the source of the scattered radiation is assumed to be a line source of length roughly equal to 1/*μ*, then for 6 MV, inverse square behavior is not expected (ignoring attenuation by the air) until *d_s_ *>> 200 m. The quantity *d_i_* in Eq. (1) is equal to *h* + 3. The dependence of Eq. (7) on *h* is not an inverse square.

**Figure 3:**
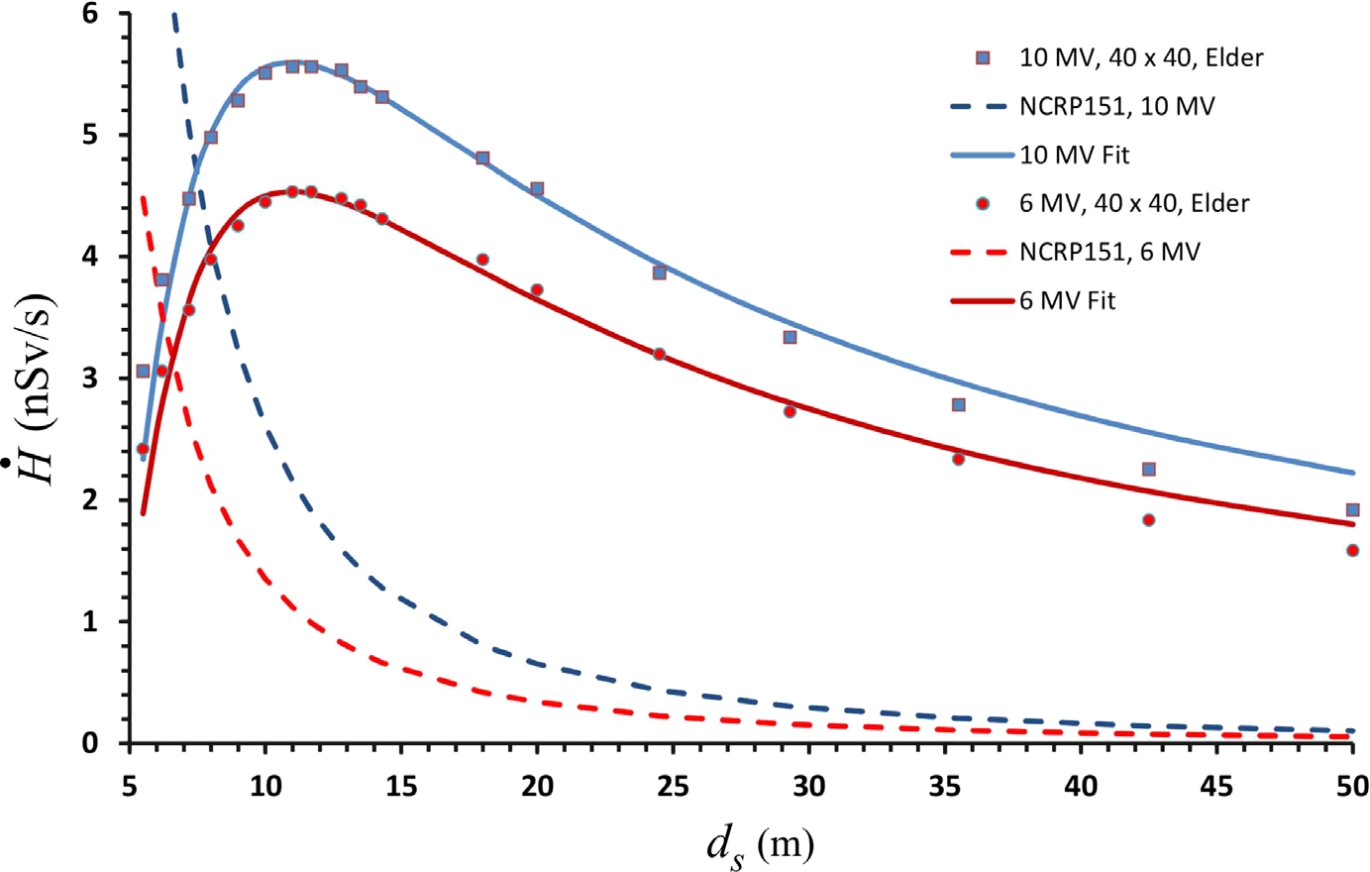
The solid curves show the fits to Eq. (7) of the skyshine dose rates measured by Elder et al. for 6 and 10 MV (40 × 40 cm^2^) as a function of the distance from the isocenter. For comparison, the dashed curves are the predictions of Eq. (1).

**Figure 4:**
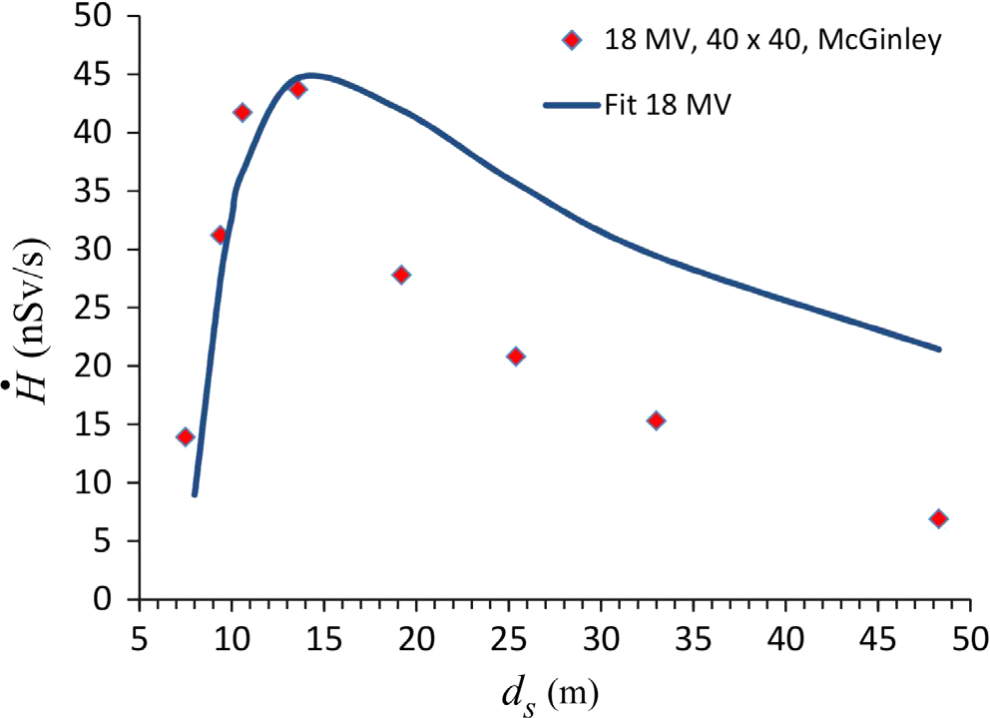
The solid curve shows the fit to Eq. (7) of the skyshine dose rates measured by McGinley for 18 MV (40 × 40 cm^2^) as a function of the distance from the isocenter.

Some of the data in Fig. 1 have been fit to equation (7) by finding values of the constant *k* that best reproduce the data. These values are listed in Table 2. Figure 3 shows the fit for the 40 × 40 cm^2^ data from the paper by Elder et al.^4^ For comparison purposes the predictions of Eq. (1) are also shown. The *k* values are 312 for 6 MV and 200 for 10 MV. The largest discrepancies (20%–25%) between the measured values and the fits occur at the shortest distance (*d_s_* = 5.5 m). This may be due to the neglect of multiple scatter in the “shadow” of the sidewall. For 5.5 m < *d_s_* < 20 m, the differences are <10%. The fit to the McGinley 18 MV data (shown in Fig. 4) is not as good, with differences as large as a factor of 2 at *d_s_* = 50 m. It was previously discussed that the McGinley data scales as $d_{s}^{-1.5}$ and therefore the measured dose rate drops off faster than Eq. (7) predicts. The reason for this is unknown. If anything, one might expect that the approximations made in deriving Eq. (7) would be best for 18 MV. This may be due in part to attenuation of the scattered photons. For *d *> *d_max_*, all measured dose rates are smaller than predicted by Eq. (7).

**Table 2:** Skyshine fitting constants.

Energy	6 MV	10 MV	18 MV
*k* (nSv/s)	312	200	160

**Figure 5:**
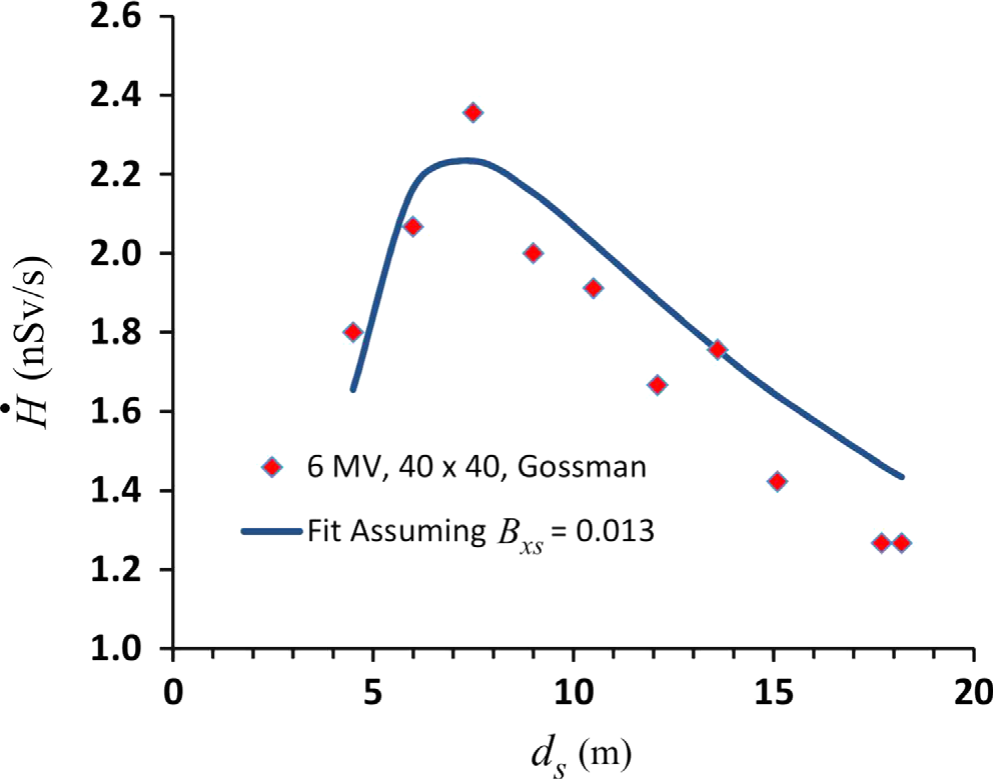
The solid curve shows the fit to Eq. (7) of the skyshine dose rates measured by Gossman et al. for 6 MV (40 × 40 cm^2^) as a function of the distance from the isocenter. A value of *B_xs_* = 0.013 has been used for the fit. This is approximately 1/3 of the value quoted by Gossman, which is based only on the concrete in the roof of the facility. The presence of 5 cm of steel would reduce the reported transmission by more than a factor of three.

As a test of Eq. (7), it can be compared with the data measured by Gossman et al for a 40 × 40 cm^2^ 6 MV beam using their quoted value of *B_xs_* = 0.038.^2^ Comparison of the measured data to the prediction of Eq. (7), reveals that the fit is almost exactly a factor of three larger at every measurement point. It was discussed previously that the value quoted for *B_xs_* is almost certainly too high. If the actual value of *B_xs_* is a third of the quoted value, the values predicted by Eq. (7) agree with the measurements to within 15%. This is shown in Fig. 5. A thickness of steel of 5 cm would more than reduce the transmission by a factor of 3. Surely concrete is not all that is in the ceiling of that facility.

Monte Carlo calculations would be helpful to confirm or modify the values of *k* in Table 2.^9^ Such calculations have been made by Kong, et al but these computations appear to have been done for 9, 15, and 21 MV beam energies and only for distances >20 m. A distance of 20 m is significantly beyond the expected value of *d_max_* for nominal room dimensions. In addition to this, these authors appear to misunderstand the meaning of Ω for photons as their definition is: “the solid angle between the source and the vertical wall.”

The only energy dependent terms in Eq. (7) are *k* and *B_xs_*. In the limit as *B_xs_* → 1 this predicts that a 6 MV beam will have approximately two times as much skyshine as an 18 MV beam (for equal field size and dose rate at isocenter). This can also be seen in Fig. 1 in which the transmission factor has been divided out. The maximum $\dot{H}$ in Fig. 1 for 6 MV is about 2.3 times larger than for 18 MV. This implies that *for lightly shielded roofs, photon skyshine should be measured at the lowest beam energy.* Photon skyshine for 6 MV is expected to dominate 18 MV skyshine for a roof with an equivalent concrete thickness of less than about 0.5 m. The physical explanation for this is, at least in part, that low energy photons are more easily scattered through large angles and that the scattered photon energy is insensitive to the primary energy.

The weekly dose equivalent corresponding to Eq. (7) is given by:

(8) $$H_{w}=1.5\times 10^{-2}\;\;k\left(WUT\right)\left(\frac{F_{0}}{400{\;\text{cm}}^{2}}\right)\frac{B_{\mathrm{xs}}}{d_{s}}\left[2{\left(1+x^{2}\right)}^{3/2}-x\left(2x^{2}+3\right)\right],$$

where $H_{w}$ is in units of μSv/week, *k* should be taken from Table 2 (in nSv/s), *W* is in units of Gy/week, *F*_0_ is in units of cm^2^ and *d_s_* is in units of meters. Let us consider the weekly dose equivalent for skyshine for a worst case scenario (6 MV, *B_xs_* = 1.0; no roof). It is assumed that *W* = 500 Gy/week, *U* = 0.25, *T* = 1/20 (outdoors with seating) and *F*_0_ = 1600 cm^2^. Nominal values of *d_w_* = 5 m and *h* = 4 m are chosen. For these parameters, the maximum dose rate is ${\dot{H}}_{w}$ ≈ 10 μSv/week at a distance of 12 m. This is about ½ of the maximum recommended permissible value for an uncontrolled area.^1^

Consider the following question. Given the scenario above, if the side wall barrier is adequately shielded so that the weekly equivalent dose rate is at an ALARA level of *P* = 10 μSv/week at a distance of 0.3 m from the side wall, will the skyshine, when added to this, exceed 20 μSv/week at any distance? Let us first consider the case in which the side wall is a primary barrier. In this instance ${\dot{H}}_{\mathrm{pri}}=P{\left[\left(d_{w}+1.3\right)/\left(d_{s}+1\right)\right]}^{2}$. Using the values listed in the previous paragraph, the total weekly dose is found to have a maximum value of about 13 μSv at a distance of about 4 m from the side wall. At a distance *d_max_* the total weekly dose is about 12 μSv. For a secondary barrier ${\dot{H}}_{\sec}=P{\left[\left(d_{w}+0.3\right)/d_{s}\right]}^{2}$, and the results are similar to the primary barrier case. We may therefore conclude that: in a worst case scenario in which there is no roof, *if the side wall is adequately shielded to an ALARA level of 10 μSv/week by itself, then it is unlikely that the total weekly photon dose will rise above 20 μSv/week at any distance.*

Under the very pessimistic assumptions of the scenario in the previous two paragraphs, the dose equivalent rate received at a distance of 1 m above the roof surface in any 1 h will be *H_h,r_* = *B_xs_ W_h_ U*/(*h* + 2)^2^ where *W_h_* is the workload for 1 h (assume *W_h_* = 800 cGy, *U = *0.25). For *h* = 4 m, and *B_xs_* = 1, *H_h,r_* = 5600 mrem. This greatly exceeds the 100 mrem in any 1 h that the US Nuclear Regulatory Commission defines as a “high radiation area” level and will therefore require signage and restricted access control in the form of an interlock or an alarm, etc.^10^ Most states follow this definition. Assuming the parameters above, a value of *B_xs_* < 0.018 is required to avoid “high radiation area” designation. This requires about 80 cm of concrete for an 18 MV beam. For 6 MV, this will result in a maximum skyshine of approximately 0.2 μSv and about 0.1 μSv for 18 MV (same *B_xs_* and therefore thicker barrier) at a distance of about 6 m from the sidewall. This is two orders of magnitude lower than the recommended maximum weekly dose value and therefore we can conclude: *if the roof is shielded sufficiently to avoid designation as a high radiation area, the photon skyshine is negligible.*

## 4 CONCLUSIONS

The formula for photon skyshine quoted in NCRP 151 [Eq. (1) in this paper] is seriously inaccurate for medical linacs and does not even make correct qualitative predictions of the dose rate. It is likely that this equation was never intended to be applied in this situation. Neutron skyshine and side scatter radiation for adjacent multistory structures are not discussed here and must be evaluated separately. The measured neutron dose equivalent rate reported in NCRP 151(table 5.2) for an 18 MV facility can exceed that for photons. It is to be noted that the NCRP151 formula for neutron skyshine is also extremely inaccurate and may be susceptible to the same type of analysis as reported here for photons.

The skyshine dose rate is directly proportional to the field area and not Ω^1.3^ Measurements should be made with the largest possible field size. Measurements of skyshine should be made at various distances to locate the maximum dose rate. The distance of the maximum dose rate from the outer surface wall depends on the ratio of the roof height above isocenter to the distance to the outer surface of the side wall from the isocenter. This distance is approximately twice the isocenter to rooftop distance for nominal room dimensions. This could preclude the use of this location for seating or an attendant booth for a parking lot. The local maximum in the dose rate occurs because the distance that the scatter has to travel to reach the observer initially *decreases* rapidly as the observer moves away from the side wall, and then begins to increase. $\dot{H}\propto 1/d_{s}$ for intermediate distances (up to about 50 m) and not $1/d_{s}^{2}$. Measurements should be made at the same height as the isocenter (1.3 m) above ground level (assuming level ground). This corresponds roughly to human waist or thorax level.

Equation (7) and (8) may be used to predict instantaneous and weekly skyshine dose rates but caution is advised due to uncertainties in the values of the fitting constant *k*. The values of this parameter reported in Table 2, depend crucially on the accuracy of *B_xs_*. The values for 6 and 10 MV are based on measurements of *B_xs_* for a 10 × 10 cm^2^ field and are presumably fairly accurate. The value of *k* for 18 MV is based on a fit to measured 18 MV data for which *B_xs_* is variously reported as 1.0 (no roof) or 0.9. As little as 2 cm of steel implies *B_xs_* = 0.66 for 18 MV radiation. This would lead to a 50% error in the derived value of *k*. Monte Carlo calculations would be very helpful to “benchmark” Eq. (7) and provide more definitive values of the fitting constant *k.* Such calculations should concentrate on distances <20 m, typical values of *h*/*d_w_*, and common beam energies of 6, 10, 15, and 18 MV. Measurements for research purposes should include a direct measurement of *B_xs._*.

It is recommended that the roof be shielded so as to avoid designation as a “high radiation area” (100 mrem in any 1 h). For nominal parameters this will require *B_xs_* ≲ 0.02. This corresponds to about 1 m of concrete for 18 MV. With this level of roof shielding, the photon skyshine is totally negligible.

If the roof does not have added shielding, it may be prudent to assume that *B_xs_* = 1.0 as a worst case scenario and use Eq. (7) and (8) to predict the dose rates. In this instance, survey measurements should be made at the lowest beam energy as well as the highest because the dose rate may be highest at the lowest energy. Under these circumstances, if the side wall is shielded to an ALARA level of 10 μSv per week, it is unlikely that the total weekly dose, including skyshine, will exceed 20 μSv at any distance from the side wall.

## CONFLICT OF INTEREST

The author declares there is no conflict of interest to disclose.

## Notes

1 This may need to be considered if there is outdoor seating or a parking area with an attendant.

2 This is *not* an attempt to make a first principles calculation of the dose rate accurate to three significant digits.

3
*d*σ/*d*Ω can be expanded to 2nd order in 1/*α*. This produces a slightly more accurate version of Eq. (7) but one that is also more complicated.
